# Impact of secondary mitral regurgitation on survival in atrial and ventricular dysfunction

**DOI:** 10.1371/journal.pone.0277385

**Published:** 2022-12-22

**Authors:** Makoto Mori, Cheryl K. Zogg, Andrea Amabile, Soraya Fereydooni, Ritu Agarwal, Gabe Weininger, Markus Krane, Lissa Sugeng, Arnar Geirsson

**Affiliations:** 1 Divison of Cardiac Surgery, Yale School of Medicine, New Haven, Connecticut, United States of America; 2 Center for Outcomes Research and Evaluation, Yale-New Haven Hospital, New Haven, Connecticut, United States of America; 3 Joint Data Analytics Team, Yale New Haven Health System, New Haven, Connecticut, United States of America; 4 Section of Cardiovascular Medicine, Department of Internal Medicine, Yale School of Medicine, New Haven, Connecticut, United States of America; 5 Yale Echocardiographic Core Laboratory, Yale-New Haven Health, New Haven, Connecticut, United States of America; BSMMU: Bangabandhu Sheikh Mujib Medical University, BANGLADESH

## Abstract

**Background:**

Natural history of atrial and ventricular secondary mitral regurgitation (SMR) is poorly understood. We compared the impact of the degree of SMR on survival between atrial and ventricular dysfunction.

**Methods:**

We conducted a retrospective cohort study of patients who underwent echocardiography in a healthcare network between 2013–2018. We compared the survival of patients with atrial and ventricular dysfunction, using propensity scores developed from differences in patient demographics and comorbidities within SMR severity strata (none, mild, moderate or severe). We fitted Cox proportional hazards models to estimate the risk-adjusted hazards of death across different severities of SMR between patients with atrial and ventricular dysfunction.

**Results:**

Of 11,987 patients included (median age 69 years [IQR 58–80]; 46% women), 6,254 (52%) had isolated atrial dysfunction, and 5,733 (48%) had ventricular dysfunction. 3,522 patients were matched from each arm using coarsened exact matching. Hazard of death in atrial dysfunction without SMR was comparable to ventricular dysfunction without SMR (HR 1.1, 95% CI 0.9–1.3). Using ventricular dysfunction without SMR as reference, hazards of death remained higher in ventricular dysfunction than in atrial dysfunction across increasing severities of SMR: mild SMR (HR 2.1, 95% CI 1.8–2.4 in ventricular dysfunction versus HR 1.7, 95%CI 1.5–2.0 in atrial dysfunction) and moderate/severe SMR (HR 2.8, 95%CI 2.4–3.4 versus HR 2.4, 95%CI 2.0–2.9).

**Conclusions:**

SMR across all severities were associated with better survival in atrial dysfunction than in ventricular dysfunction, though the magnitude of the diminishing survival were similar between atrial and ventricular dysfunction in increasing severity of SMRs.

## Background

Atrial functional mitral regurgitation (AFMR) is a form of secondary mitral regurgitation (SMR) increasingly gaining recognition [1]. AFMR is thought to occur secondary to dilated and dysfunctional atrium in the absence of ventricular dysfunction [2]. This contrasts the SMR of ventricular etiology commonly seen in ischemic cardiomyopathy and dilated cardiomyopathy. While the different etiologies and potentially effective treatments are recognized between the two subtypes of SMR [3, 4], the natural history of patients with different severities of SMR related to atrial dysfunction has not been well described beyond small single-center series [5, 6]. Understanding the difference in survival between comparable severities of SMR in atrial versus ventricular dysfunction may further inform the timing of surveillance and potential therapeutic options [4].

Although the etiology of SMR in patients with isolated atrial dysfunction differs from SMR resulting from ventricular dysfunction, current guidelines do not clearly distinguish management [7]. In AFMR, insufficient leaflet remodeling in response to the annular dilatation appears to be the driver of the disease [3] and SMR may temporarily improve after correction of atrial fibrillation [8, 9]. Therefore, the optimal surgical approach and threshold for intervention in AFMR may differ from ventricular SMR [10–12]. Understanding the potential difference in the impact of SMR on survival in patients with atrial dysfunction relative to ventricular dysfunction may help guide the threshold for intervention.

Leveraging a large echocardiogram database of a health care system, we aimed to compare the impact of different severities of SMR on survival between those with atrial and ventricular dysfunctions.

## Methods

### Data source and patient population

This retrospective cohort study was conducted at Yale-New Haven Health, a large healthcare network in the United States, encompassing 5 acute care hospitals in academic and community settings and over 120 outpatient clinics at satellite locations throughout Connecticut [13]. The system-wide electronic medical record database was queried to identify patients age ≥ 18 years who received complete (as opposed to focused exam) transthoracic echocardiogram for any indication between July 1, 2013, and October 31, 2018, either during inpatient or outpatient encounters. These included echocardiograms obtained from 32 sites.

Structured data fields on echocardiographic parameters and full text of the cardiology attendings’ echocardiogram reads were extracted. We identified patients with isolated atrial dysfunction or ventricular dysfunction using the following definitions: isolated atrial dysfunction was defined as enlarged left atrium (LA dimension index >34 mm/m^2^) [14] with LVEF≥55% or history of atrial fibrillation or flutter with LVEF ≥55% based on prior studies [15]. Ventricular dysfunction was defined as those with LVEF <55% regardless of atrial dimension or atrial arrhythmia histories. We adopted this definition while recognizing that prior studies have variably defined atrial dysfunction [16]. LVEF was estimated using 2D or 3D volumetric tracing. We refer to SMR as MR of non-primary etiology encompassing ventricular or atrial dysfunction.

**Fig 1:**
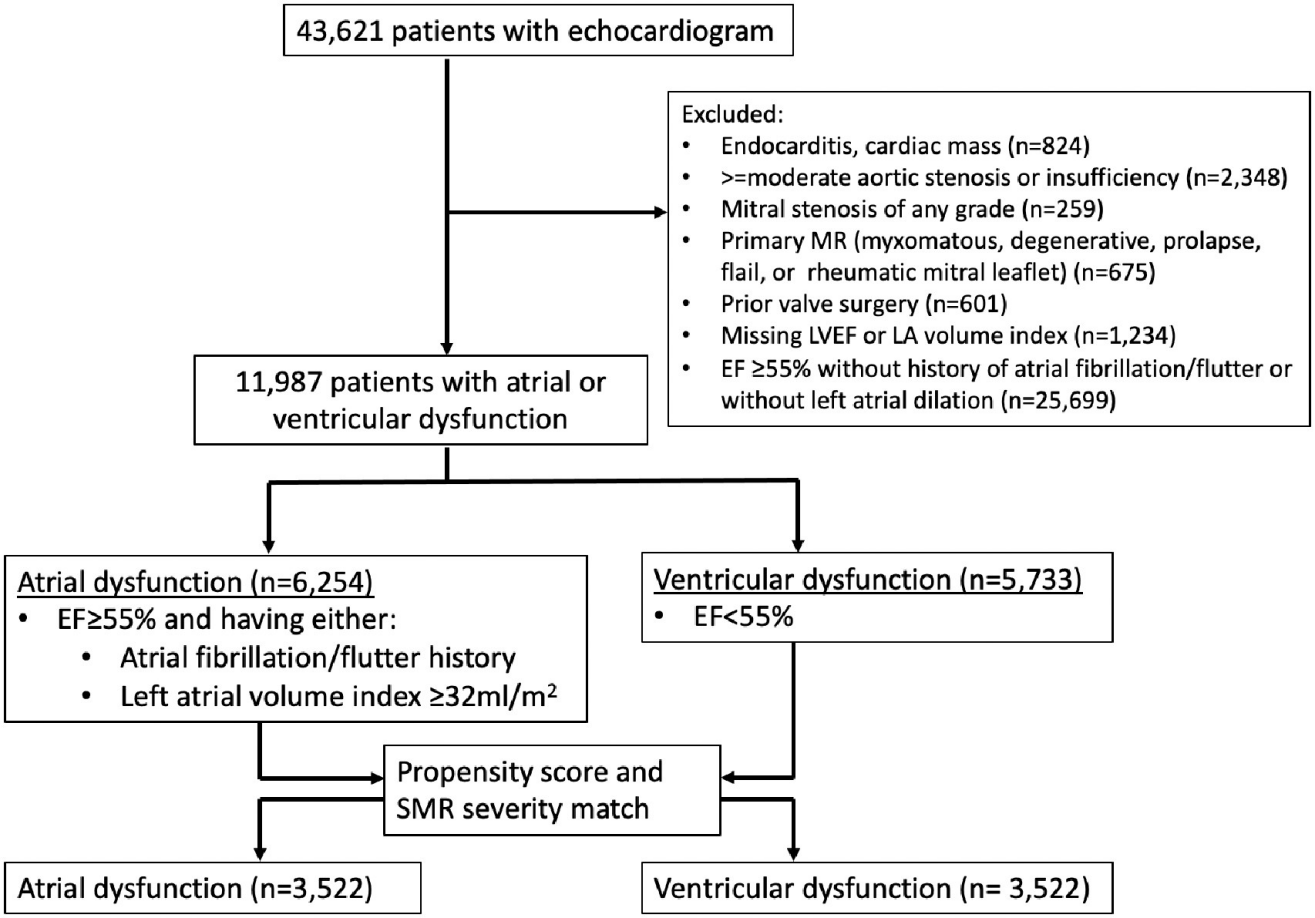
CONSORT-style diagram. The figure shows exclusion criteria and the definition of exposure groups. LA = left atrial; EF = ejection fraction; MR = mitral regurgitation.

We restricted the cohort to those without significant concomitant valvular pathologies and MR of primary etiologies by the following exclusion criteria: those with primary MR of any severity (rheumatic, prolapsed, degenerative, or myxomatous findings), mitral stenosis of any severity, greater-than-mild aortic stenosis or aortic regurgitation, those with findings of vegetation or intracardiac mass or thrombus. Patients with prosthetic valves were also excluded (Fig 1). Additionally, patients with missing ejection fraction or left atrial dimension index were excluded. In patients with multiple echocardiograms, the study with the earliest date was analyzed. These criteria yielded 11,987 echocardiograms obtained on unique patients with either atrial or ventricular dysfunction, with secondary MR grades ranging from none to severe.

The Institutional Review Board at Yale University approved this study, and individual consent was waived for the minimal risk nature of the study.

### Outcome

The outcome of the study was cumulative survival since the time of echocardiogram. Patient data were linked to the Connecticut Vital Statistics data curated by the Connecticut State Department of Public Health to ascertain the date of death. The state’s vital statistics record captures deaths of all Connecticut residents, whether the death occurred in or out of the state. The last date of follow-up available via the vital statistics data was October 15, 2019.

### Echocardiographic and patient characteristics, missing values

Demographic variables extracted from the medical records system were age at the time of echocardiogram, sex, and race. Comorbidities were selected based on prior studies and identified via the patient’s past medical history that were grouped based on the corresponding *International Classification of Diseases 10*^*th*^ *revision*, *Clinical Modification* (ICD-10-CM) codes [17]. The extracted comorbidities were updated and verified by the care provider at each clinical encounter and were distinct from those used for billing purposes. The comorbidities were: heart failure, diabetes, hypertension, myocardial infarction, peripheral vascular disease, prior coronary artery bypass graft surgery, cerebrovascular disease, chronic pulmonary obstructive disease, liver disease, renal failure, iron-deficiency anemia, rheumatoid disease, peptic ulcer disease, dementia, depression, cancer, substance use, and acquired immunodeficiency syndrome (S1 Table) [17]. In addition to the severity of the valvular disease, the left ventricular ejection fraction and left atrial volume index was extracted from the echocardiogram report. This was missing in 1,234 patients, who were excluded. Patient age, sex, and race had no missing values. Comorbidity variables defined via ICD-10 codes did not have any missing values as the absence of ICD-10 codes corresponding to the condition of interest was defined as the absence of a condition consistent with methodologies adopted by existing studies.

### Interval mitral valve surgery, transcatheter mitral valve interventions, and ablations

We linked the echocardiogram database with the institutional Society of Thoracic Surgeons Adult Cardiac Surgery Database and the list of cardiovascular procedures that the patient underwent within our health system since the time of the index echocardiogram and October 15, 2019. Mitral valve surgery (mitral valve replacement or repair) or mitral valve intervention (transcatheter edge-to-edge repair or transcatheter mitral valve replacement) was treated as the time-varying covariate in the Cox proportional hazard models. Similarly, catheter-based ablations performed for atrial fibrillation or flutter were identified and matched.

### Statistical analysis

Continuous variables were reported as medians and interquartile ranges (IQR). Categorical variables were expressed as frequencies and percentages. Differences in demographic variables between patients with atrial and dysfunction were compared using Wilcoxon rank-sum tests for non-normally distributed continuous variables and the chi-squared tests for categorical variables.

Kaplan-Meier plots and corresponding log-rank tests were calculated to evaluate unadjusted and matched survival estimates. The study aimed to compare the potential impact of the degree of SMR on survival between patients with isolated atrial dysfunction and patients with ventricular dysfunction. To account for potential confounding between survival and atrial or ventricular dysfunction, matching between isolated atrial and ventricular dysfunction groups was performed by first estimating propensity scores from a multivariable logistic regression model using the covariates listed below. The groups were then matched with a 1:1 coarsened exact nearest-neighbor matching method without replacement using the propensity score and matching the groups exactly on the severity of SMRs [18]. Observations without nearest neighbor match were discarded. This method was used to balance the observed patient characteristics between the two groups across different severity strata of SMRs. The quality of matching was evaluated using the Love plot depicting the standard mean difference of the groups in each covariate and the density plot of the propensity score. We defined the standard mean difference <0.1 as the threshold for balanced matching [19]. We fitted a bivariate Cox proportional hazard model on the matched dataset, including both ventricular and atrial dysfunction patients, to assess the hazard of time-to-death associated with the severity of SMR occurring with atrial or ventricular dysfunction. Hazard ratios for each combination of ventricular versus atrial dysfunction and SMR severities were calculated using the group with ventricular dysfunction without SMR as the reference group.

The propensity score was calculated from the following covariates: atrial or ventricular dysfunction, age, sex, race, inpatient status (in vs. outpatient), heart failure, diabetes, hypertension, myocardial infarction, peripheral vascular disease, prior coronary artery bypass graft surgery, cerebrovascular disease, chronic pulmonary obstructive disease, liver disease, renal failure, iron-deficiency anemia, rheumatoid disease, peptic ulcer disease, dementia, depression, cancer, substance use, and acquired immunodeficiency syndrome. Additionally, mitral valve surgery or intervention and ablation for atrial arrhythmias were matched on time from echo to intervention.

As a sensitivity analysis to assess the differential impact of SMR on the hazard of death between patients with atrial and ventricular dysfunction, Cox models were fitted separately for atrial and ventricular dysfunction groups using the demographic, comorbidity, and SMR severity as time-independent covariates and mitral valve surgery or intervention as a time-dependent covariate. The proportional hazard assumption was evaluated for the MR severity and atrial or ventricular dysfunction using the survival plot, which satisfied the assumption. Because the coefficients of different models cannot be directly compared for the potentially varying residuals [20], we estimated the number of deaths during the constant follow-up duration under the simulated condition of every patient having no SMR, mild SMR, and moderate or severe SMR. This was done separately for patients with atrial dysfunction and ventricular dysfunction. By comparing the percent changes in the number of deaths under all patients having no SMR to all patients having severe SMR, we estimated the impact of increasing severity of SMR on the hazard of death. We characterized the differential impact of SMR between patients with atrial and ventricular dysfunction by comparing the estimated percent increase in the number of deaths as the severity of SMR increased.

A two-tailed *p* < 0.05 was considered significant. All analyses were conducted with RStudio 1.3.1073 (Rstudio, PBC, Boston, MA) with packages *dplyr*, *gtsummary*, *matchit*, *survival*, and Python (Python Software Foundation) packages *pandas* and *regex*.

This study was conducted under IRB approval (protocol ID: 2000028791, approved by Yale IRES/IRB on date: 9/16/2020).

## Results

Among the 11,987 patients, there were 6,254 (52.2%) patients with isolated atrial dysfunction and 5,733 (47.8%) patients with ventricular dysfunction. The median age of the cohort was 69 (IQR 58, 80) years with 46% being male and 80% of echocardiogram performed in an inpatient setting. Patients with atrial dysfunction were older and more frequently female. Compared with patients with ventricular dysfunction, some atherosclerotic comorbidities were less common in patients with atrial dysfunction, such as the history of diabetes, peripheral vascular disease, prior CABG, and myocardial infarction. Liver disease, cancer, and substance use were more common in patients with atrial dysfunction. Moderate or severe MR was more frequently present in patients with ventricular dysfunction (757 [12%] versus 1,366 [24%], p<0.001) (Table 1).

**Table 1:** Patient characteristics by atrial and ventricular dysfunction.

Variables	Overall (N = 11,987)	Atrial dysfunction (N = 6,254)	Ventricular dysfunction (N = 5,733)	P
Age (year)	69 (58, 80)	70 (59, 81)	67 (56, 79)	<0.001
Female	5,556 (46%)	3,418 (55%)	2,138 (37%)	<0.001
*Race*				<0.001
White	8,906 (74%)	4,743 (76%)	4,163 (73%)	
Black	2,021 (17%)	952 (15%)	1,069 (19%)	
Other	1,060 (8.8%)	559 (8.9%)	501 (8.7%)	
Body surface area (m^2^)	1.92 (1.72, 2.13)	1.89 (1.69, 2.11)	1.94 (1.75, 2.15)	<0.001
Hypertension	8,488 (71%)	4,480 (72%)	4,008 (70%)	0.038
Diabetes	3,729 (31%)	1,796 (29%)	1,933 (34%)	<0.001
Prior CABG	223 (1.9%)	64 (1.0%)	159 (2.8%)	<0.001
Prior PCI	115 (1.0%)	36 (0.6%)	79 (1.4%)	<0.001
Heart failure	2,621 (22%)	895 (14%)	1,726 (30%)	<0.001
Peripheral vascular disease	1,689 (14%)	691 (11%)	998 (17%)	<0.001
Myocardial infarction	1,668 (14%)	464 (7.4%)	1,204 (21%)	<0.001
Atrial fibrillation/flutter	3,438 (29%)	1,987 (32%)	1,451 (25%)	<0.001
Cerebrovascular disease	2,161 (18%)	1,216 (19%)	945 (16%)	<0.001
Renal failure	1,811 (15%)	898 (14%)	913 (16%)	0.017
Liver disease	663 (5.5%)	469 (7.5%)	194 (3.4%)	<0.001
Iron deficiency anemia	177 (1.5%)	103 (1.6%)	74 (1.3%)	0.11
Peptic ulcer disease	246 (2.1%)	138 (2.2%)	108 (1.9%)	0.2
Rheumatoid disease	448 (3.7%)	257 (4.1%)	191 (3.3%)	0.025
Dementia	565 (4.7%)	317 (5.1%)	248 (4.3%)	0.055
Depression	1,769 (15%)	992 (16%)	777 (14%)	<0.001
Cancer	2,871 (24%)	1,629 (26%)	1,242 (22%)	<0.001
Substance use	764 (6.4%)	433 (6.9%)	331 (5.8%)	0.01
AIDS	120 (1.0%)	62 (1.0%)	58 (1.0%)	>0.9
*Echo data*				
Inpatient setting	9,214 (80%)	4,540 (77%)	4,674 (85%)	<0.001
Setting unknown	536	324	212	
Left atrial volume index	38 (32, 48)	39 (34, 48)	36 (27, 48)	<0.001
*Mitral regurgitation*				<0.001
None/trace	4,118 (34%)	2,428 (39%)	1,690 (29%)	
Mild	5,746 (48%)	3,069 (49%)	2,677 (47%)	
Moderate/severe	2,123 (18%)	757 (12%)	1,366 (24%)	
*Tricuspid regurgitation*				<0.001
None/trace	6,362 (53%)	3,364 (54%)	2,998 (52%)	
Mild	3,495 (29%)	1,865 (30%)	1,630 (28%)	
Moderate/severe	2,130 (18%)	1,025 (16%)	1,105 (19%)	
Ejection fraction (%)	57 (46, 64)	64 (60, 68)	45 (35, 52)	<0.001
LVIDd (cm)	4.79 (4.30, 5.29)	4.59 (4.17, 4.99)	5.08 (4.55, 5.65)	<0.001
Missing	4,419	2,202	2,217	
LVIDs (cm)	3.26 (2.82, 3.89)	2.96 (2.61, 3.31)	3.84 (3.27, 4.55)	<0.001
Missing	4,438	2,212	2,226	
*Surgery/interventions during follow-up*				
Mitral valve replacement	141 (1.2%)	53 (0.8%)	88 (1.5%)	<0.001
Transcatheter edge-to-edge repair	6 (<0.1%)	0 (0%)	6 (0.1%)	0.012
Atrial fibrillation/flutter ablation	237 (2.0%)	119 (1.9%)	118 (2.1%)	0.5

Continuous variables are expressed in terms of median (interquartile range) and categorical variables are expressed as N (%). CABG = coronary artery bypass graft; PCI = percutaneous coronary intervention; AIDS = acquired immunodeficiency syndrome. LVIDd = left ventricular internal diameter (end-diastolic); LVIDs = left ventricular internal diameter (end-systolic); TMVR = transcatheter mitral valve replacement.

**Fig 2:**
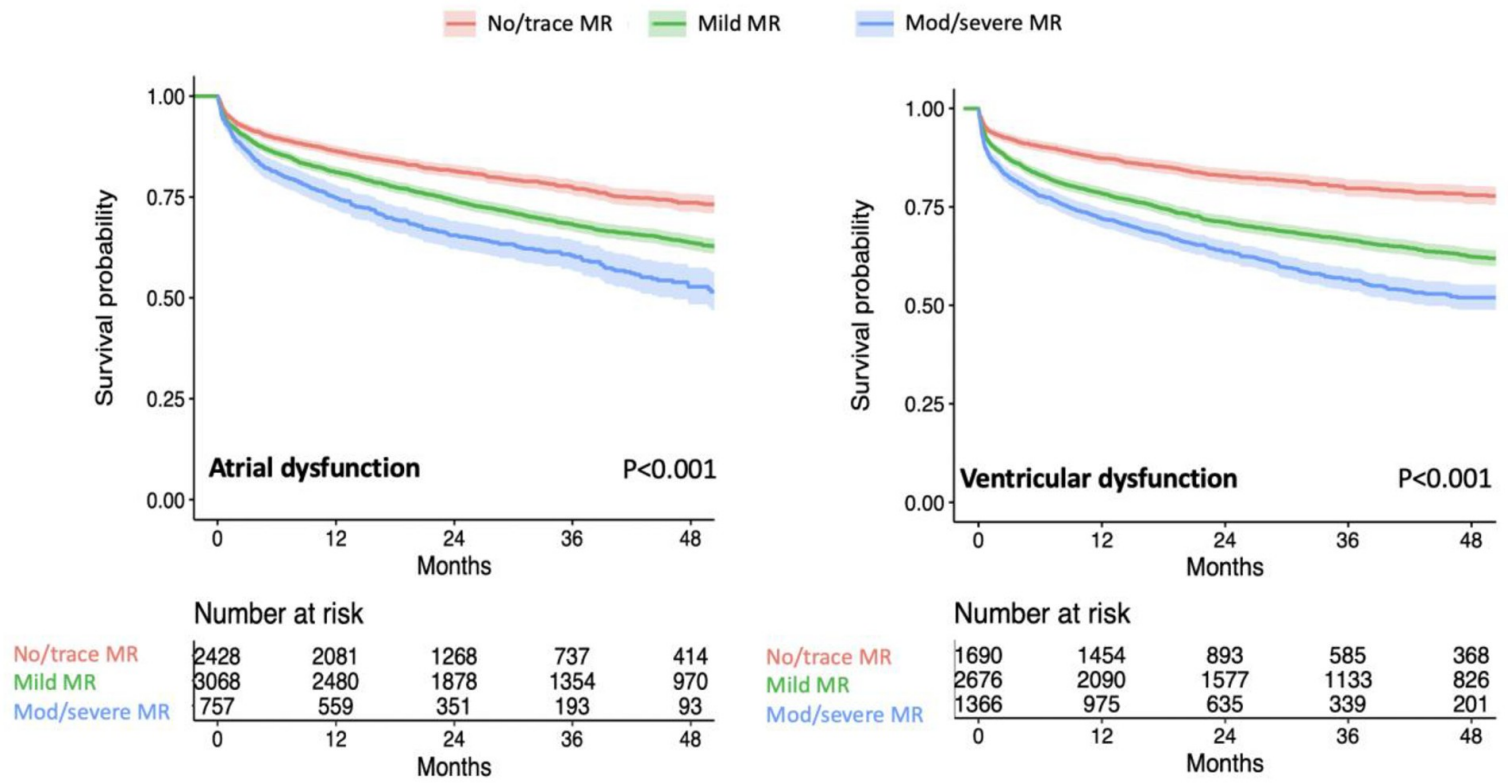
Unadjusted survival curves by different severities of mitral regurgitation. The figure shows Kaplan-Meier curves of patients with no or trace mitral regurgitation (MR) (red), mild MR (green), and moderate or severe MR (blue), stratified by patients with atrial dysfunction (left) and patients with ventricular dysfunction (right).

Among patients with isolated atrial dysfunction, there were 2,428 (39%) with no or trace SMR, 3,069 (49%) with mild SMR, and 757 (12%) with moderate or severe SMR. Among patients with ventricular dysfunction, there were 1,690 (29%) with no or trace SMR, 2,677 (47%) with mild SMR, and 1,366 (24%) with moderate or severe SMR. Patients with a more severe form of SMR in both the atrial and ventricular dysfunction groups were older, with a higher prevalence of atrial fibrillation or flutter and severer coexisting tricuspid regurgitation. In atrial dysfunction, mild or greater SMR occurred more commonly in women while it occurred more commonly in men in ventricular dysfunction (Table 2 for atrial dysfunction, Table 3 for ventricular dysfunction). Unadjusted survival was lower in patients with a higher grade of SMR in both ventricular and atrial dysfunctions (Fig 2).

**Table 2:** Characteristics of patients with atrial dysfunction by the SMR severity.

Characteristic	None/trace MR (N = 2,428)	Mild MR (N = 3,069)	Moderate/severe MR (N = 757)	P
Age (year)	64 (55, 74)	73 (62, 83)	80 (69, 88)	<0.001
Female	1,128 (46%)	1,757 (57%)	533 (70%)	<0.001
*Race*				<0.001
White	1,754 (72%)	2,396 (78%)	593 (78%)	
Black	415 (17%)	432 (14%)	105 (14%)	
Other	259 (11%)	241 (7.9%)	59 (7.8%)	
Hypertension	1,648 (68%)	2,243 (73%)	589 (78%)	<0.001
Diabetes	733 (30%)	879 (29%)	184 (24%)	0.008
Prior CABG	15 (0.6%)	39 (1.3%)	10 (1.3%)	0.04
Heart failure	217 (8.9%)	487 (16%)	191 (25%)	<0.001
Peripheral vascular disease	220 (9.1%)	369 (12%)	102 (13%)	<0.001
Myocardial infarction	177 (7.3%)	224 (7.3%)	63 (8.3%)	0.6
Atrial fibrillation/flutter	623 (26%)	1,074 (35%)	290 (38%)	<0.001
Cerebrovascular disease	441 (18%)	639 (21%)	136 (18%)	0.026
Renal failure	295 (12%)	462 (15%)	141 (19%)	<0.001
Liver disease	182 (7.5%)	246 (8.0%)	41 (5.4%)	0.052
Iron deficiency anemia	35 (1.4%)	54 (1.8%)	14 (1.8%)	0.6
Peptic ulcer disease	43 (1.8%)	84 (2.7%)	11 (1.5%)	0.017
Rheumatoid disease	94 (3.9%)	127 (4.1%)	36 (4.8%)	0.6
Dementia	76 (3.1%)	197 (6.4%)	44 (5.8%)	<0.001
Depression	408 (17%)	499 (16%)	85 (11%)	<0.001
Cancer	577 (24%)	853 (28%)	199 (26%)	0.003
Substance use	197 (8.1%)	195 (6.4%)	41 (5.4%)	0.008
AIDS	17 (0.7%)	35 (1.1%)	10 (1.3%)	0.2
*Echo data*				
Inpatient setting	1,649 (72%)	2,305 (79%)	586 (82%)	<0.001
Setting unknown	142	141	41	
Left atrial volume index	37 (33, 43)	40 (35, 49)	48 (39, 61)	<0.001
*Tricuspid regurgitation*				<0.001
None/trace	2,073 (85%)	1,118 (36%)	173 (23%)	
Mild	291 (12%)	1,326 (43%)	248 (33%)	
Moderate/severe	64 (2.6%)	625 (20%)	336 (44%)	
Ejection fraction (%)	64.0 (60.0, 68.0)	64.0 (60.0, 68.0)	63.0 (60.0, 67.0)	0.05
LVIDd (cm)	4.64 (4.20, 5.04)	4.56 (4.15, 4.95)	4.53 (4.08, 4.94)	<0.001
Missing	532	1,493	177	
LVIDs (cm)	2.99 (2.64, 3.32)	2.94 (2.60, 3.29)	2.93 (2.57, 3.31)	0.063
Missing	537	1,497	178	
*Surgery/interventions during follow-up*				
Mitral valve surgery/TMVR	6 (0.2%)	21 (0.7%)	26 (3.4%)	<0.001
Transcatheter edge-to-edge repair	0 (0%)	0 (0%)	0 (0%)	
Atrial fibrillation/flutter ablation	53 (2.2%)	55 (1.8%)	11 (1.5%)	0.4

Continuous variables are expressed in terms of median (interquartile range) and categorical variables are expressed as N (%). CABG = coronary artery bypass graft; AIDS = acquired immunodeficiency syndrome. LVIDd = left ventricular internal diameter (end-diastolic); LVIDs = left ventricular internal diameter (end-systolic).

**Table 3:** Characteristics of patients with ventricular dysfunction by the SMR severity.

Characteristic	None/trace MR (N = 1,690)	Mild MR (N = 2,677)	Moderate/severe MR (N = 1,366)	P
Age (year)	60 (51, 70)	69 (59, 80)	74 (61, 84)	<0.001
Female	524 (31%)	1,022 (38%)	592 (43%)	<0.001
*Race*				0.13
White	1,216 (72%)	1,973 (74%)	974 (71%)	
Black	326 (19%)	463 (17%)	280 (20%)	
Other	148 (8.8%)	241 (9.0%)	112 (8.2%)	
Hypertension	1,099 (65%)	1,899 (71%)	1,010 (74%)	<0.001
Diabetes	535 (32%)	929 (35%)	469 (34%)	0.1
Prior CABG	25 (1.5%)	90 (3.4%)	44 (3.2%)	<0.001
Heart failure	282 (17%)	815 (30%)	629 (46%)	<0.001
Peripheral vascular disease	209 (12%)	505 (19%)	284 (21%)	<0.001
Myocardial infarction	379 (22%)	551 (21%)	274 (20%)	0.2
Atrial fibrillation/flutter	232 (14%)	759 (28%)	460 (34%)	<0.001
Cerebrovascular disease	222 (13%)	482 (18%)	241 (18%)	<0.001
Renal failure	181 (11%)	457 (17%)	275 (20%)	<0.001
Liver disease	52 (3.1%)	89 (3.3%)	53 (3.9%)	0.5
Iron deficiency anemia	9 (0.5%)	41 (1.5%)	24 (1.8%)	0.004
Peptic ulcer disease	29 (1.7%)	47 (1.8%)	32 (2.3%)	0.4
Rheumatoid disease	51 (3.0%)	90 (3.4%)	50 (3.7%)	0.6
Dementia	46 (2.7%)	131 (4.9%)	71 (5.2%)	<0.001
Depression	250 (15%)	370 (14%)	157 (11%)	0.026
Cancer	347 (21%)	619 (23%)	276 (20%)	0.042
Substance use	124 (7.3%)	145 (5.4%)	62 (4.5%)	0.002
AIDS	22 (1.3%)	17 (0.6%)	19 (1.4%)	0.028
*Echo data*				
Inpatient setting	1,278 (80%)	2,210 (85%)	1,186 (89%)	<0.001
Setting unknown	85	91	36	
Left atrial volume index	28 (22, 36)	37 (28, 47)	49 (39, 62)	<0.001
*Tricuspid regurgitation*				<0.001
None/trace	1,481 (88%)	1,152 (43%)	365 (27%)	
Mild	168 (9.9%)	1,058 (40%)	404 (30%)	
Moderate/severe	41 (2.4%)	467 (17%)	597 (44%)	
Ejection fraction (%)	50 (43, 54)	45 (35, 52)	35 (25, 46)	<0.001
LVIDd (cm)	4.93 (4.43, 5.42)	5.04 (4.54, 5.62)	5.39 (4.82, 6.05)	<0.001
Missing	460	1,346	411	
LVIDs (cm)	3.53 (3.13, 4.07)	3.87 (3.26, 4.50)	4.34 (3.67, 5.15)	<0.001
Missing	465	1,348	413	
*Surgery/interventions during follow-up*				
Mitral valve surgery/TMVR	12 (0.7%)	33 (1.2%)	43 (3.1%)	<0.001
Transcatheter edge-to-edge repair	0 (0%)	1 (<0.1%)	5 (0.4%)	0.004
Atrial fibrillation/flutter ablation	33 (2.0%)	57 (2.1%)	28 (2.0%)	>0.9

Continuous variables are expressed in terms of median (interquartile range) and categorical variables are expressed as N (%). CABG = coronary artery bypass graft; AIDS = acquired immunodeficiency syndrome. LVIDd = left ventricular internal diameter (end-diastolic); LVIDs = left ventricular internal diameter (end-systolic).

Matching yielded 7,044 patients (3,522 in each group). Patient characteristics were balanced except for the variables not used to generate propensity scores, which were ejection fraction and left ventricular dimensions (Table 4). The distribution of the propensity score before and after matching (S1 Fig) and the standardized mean difference for each covariate (S2 Fig) indicated that the groups were matched well.

**Table 4:** Patient characteristics of matched cohort.

Variables	Atrial dysfunction (N = 3,522)	Ventricular dysfunction (N = 3,522)
Age (year)	70 (59, 81)	70 (58, 81)
Female	1,586 (45%)	1,662 (47%)
*Race*		
White	2,634 (75%)	2,629 (75%)
Black	574 (16%)	589 (17%)
Other	314 (8.9%)	304 (8.6%)
Hypertension	2,493 (71%)	2,509 (71%)
Diabetes	1,096 (31%)	1,103 (31%)
Prior CABG	57 (1.6%)	58 (1.6%)
Heart failure	722 (20%)	778 (22%)
Peripheral vascular disease	482 (14%)	487 (14%)
Myocardial infarction	413 (12%)	353 (10%)
Atrial fibrillation/flutter	1,092 (31%)	1,085 (31%)
Cerebrovascular disease	648 (18%)	646 (18%)
Renal failure	536 (15%)	563 (16%)
Liver disease	159 (4.5%)	161 (4.6%)
Iron deficiency anemia	53 (1.5%)	49 (1.4%)
Peptic ulcer disease	68 (1.9%)	69 (2.0%)
Rheumatoid disease	129 (3.7%)	135 (3.8%)
Dementia	190 (5.4%)	197 (5.6%)
Depression	512 (15%)	528 (15%)
Cancer	813 (23%)	828 (24%)
Substance use	233 (6.6%)	224 (6.4%)
AIDS	34 (1.0%)	41 (1.2%)
*Echo data*		
Inpatient setting	2,856 (81%)	2,842 (81%)
Left atrial volume index	39 (34, 47)	37 (28, 49)
*Mitral regurgitation*		
None/trace	1,098 (31%)	1,098 (31%)
Mild	1,782 (51%)	1,782 (51%)
Moderate/severe	642 (18%)	642 (18%)
*Tricuspid regurgitation*		
None/trace	1,782 (51%)	1,710 (49%)
Mild	1,066 (30%)	1,150 (33%)
Moderate/severe	674 (19%)	662 (19%)
Ejection fraction (%)	64 (60, 68)	47 (35, 53)
LVIDd (cm)	4.62 (4.19, 5.02)	4.93 (4.42, 5.48)
Missing	1,315	1,766
LVIDs (cm)	3.00 (2.64, 3.36)	3.64 (3.15, 4.26)
Missing	1,322	1,774
Mitral valve surgery/TMVR	40 (1.1%)	42 (1.2%)
Transcatheter edge-to-edge repair	0 (0%)	2 (<0.1%)
Atrial fibrillation/flutter ablation	78 (2.2%)	78 (2.2%)

Continuous variables are expressed in terms of median (interquartile range) and categorical variables are expressed as N (%). CABG = coronary artery bypass graft; AIDS = acquired immunodeficiency syndrome. LVIDd = left ventricular internal diameter (end-diastolic); LVIDs = left ventricular internal diameter (end-systolic); TMVR = transcatheter mitral valve replacement.

**Fig 3:**
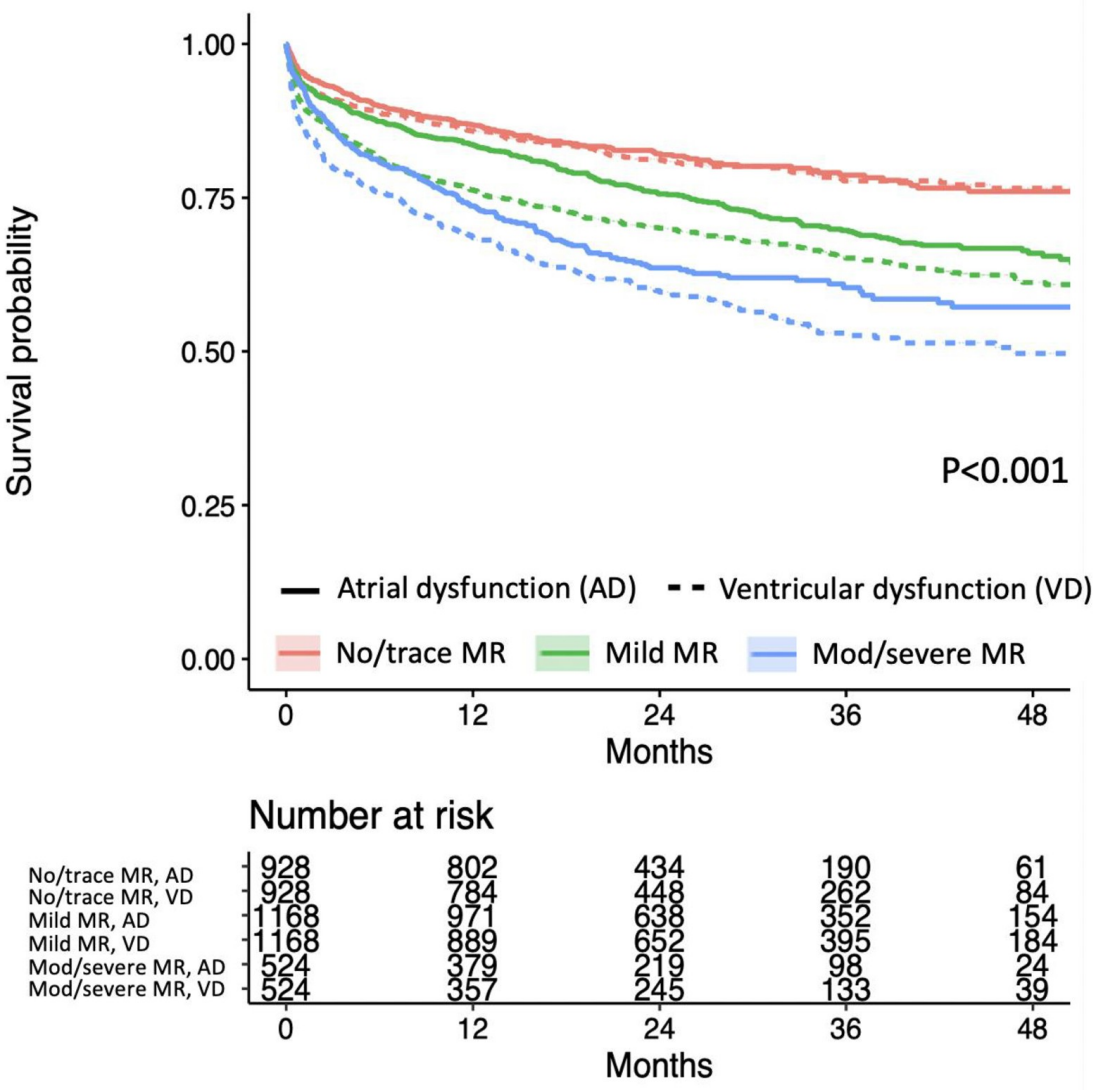
Matched group survival curves by different severities of mitral regurgitation. The figure shows Kaplan-Meier curves of patients with no or trace mitral regurgitation (MR) (red), mild MR (green), and moderate or severe MR (blue), stratified by patients with atrial dysfunction (solid) and patients with ventricular dysfunction (dotted) in the cohort matched by the propensity score and MR severity.

**Fig 4:**
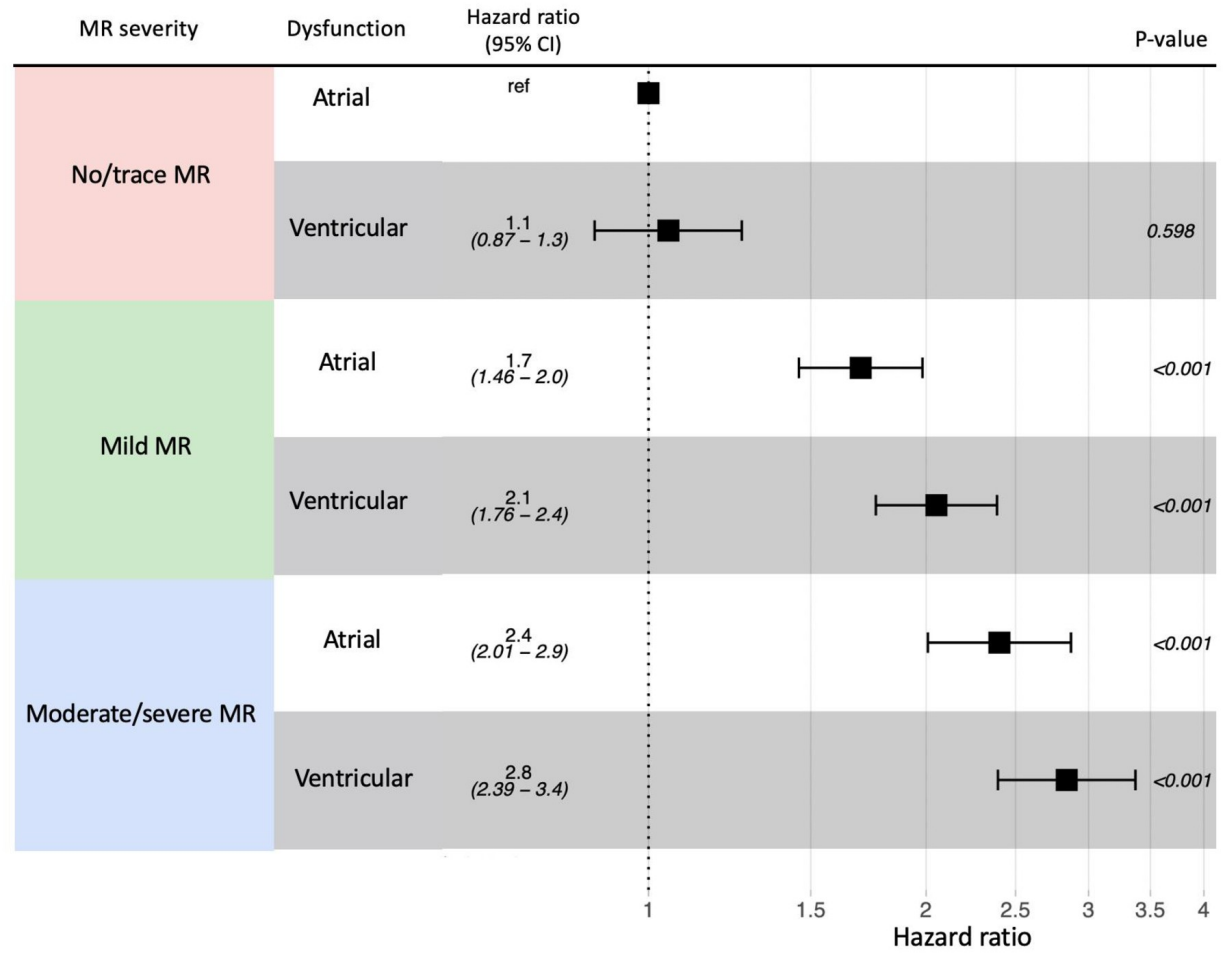
Hazard of death associated with MR severity and atrial or ventricular dysfunction in matched group. The figure shows increasing hazard of death with increasing mitral regurgitation (MR) severity. Within the same MR severity, ventricular dysfunction had higher hazard of death than in atrial dysfunction, with no or trace atrial MR as the reference (ref). CI = confidence interval.

Survival curves of the matched group demonstrated comparable survival between atrial and ventricular dysfunction with no or trace SMR. In contrast, the survival was worse in ventricular dysfunction than in atrial dysfunction in patients with mild, moderate, or severe SMR (Fig 3). Cox proportional hazard model fitted on the matched group also demonstrated that the hazard of death was not significantly different between atrial and ventricular dysfunctions in patients with no or trace SMR while the hazard was higher in patients with ventricular dysfunction in mild, moderate, and severe SMR than in patients with atrial dysfunction (Fig 4).

The sensitivity analysis using the Cox proportional hazards model also demonstrated an increasing number of expected deaths with increasing severity of SMR in both atrial and ventricular dysfunctions. The magnitude of increase with a more severe SMR was larger in ventricular dysfunction than in atrial dysfunction (S2 Table).

## Discussion

Understanding the difference in the prognosis of SMR between those related to atrial versus ventricular dysfunction is important considering the variable treatment response to interventions in patients with functional MR [5, 21, 22]. In a cohort of patients with echocardiograms performed in academic and community settings, we demonstrated that across comparable severities of MR, patients with both atrial and ventricular dysfunction portended worse survival with higher degrees of SMR. Additionally, atrial dysfunction had better survival than patients with ventricular dysfunction within comparable strata of SMR severity. These findings suggest that more severe SMR could carry a higher burden in ventricular dysfunction relative to atrial dysfunction. Different prognosis in the two different types of SMR warrants further investigation to determine the optimal threshold for intervention in each type of SMR.

This study adds to the current literature in several ways. First, the prognostic significance of SMR related to atrial and ventricular dysfunctions has been suspected [23] but direct comparisons have been limited only to small studies with an inconclusive characterization of survivals [24]. Clinical characteristics and optimal treatment approaches for MR related to ventricular dysfunction are well characterized by studies focusing on SMR occurring in the setting of ischemic cardiomyopathy [12, 25]. However, the outcomes of SMR related to atrial dysfunction remained elusive, especially in direct comparisons to those in SMR associated with ventricular dysfunction. Perhaps consequently, the current guidelines do not distinguish the treatment approach between the two types of SMR [26]. The current surveillance recommendation may be inadequate for milder forms of SMR and does not distinguish atrial from ventricular SMR [27].

A single-center study reported superior survival in AFMR compared with ventricular MR in patients who underwent mitral valve operation [5], but how such findings may translate into the broader cohort of patients with MR before the selection for surgical intervention had been unclear. Our study of patients who underwent echocardiograms in the community and academic sites demonstrated that SMR related to atrial and ventricular dysfunctions differ in patient characteristics and survival. However, both forms of SMR were associated with an increased risk of death compared to patients without SMR, supporting the notion that both forms of severe SMR require close monitoring and treatment. The observed higher hazards of death associated with mild SMR, which has been thought of as a relatively benign entity, is corroborated by prior studies demonstrating increased morality risk associated with mild SMR [27, 28].

The optimal treatment approach in terms of mitral valve intervention likely relates to the mechanistic working of MR and their prognostic significance. The simple annular dilatation alone may not explain the majority of MR occurring in the setting of atrial dysfunction. Instead, some studies have suspected it is the discrepant loading condition between LA and LV that results in malcoaptation. In such settings, a simple annuloplasty alone may not be beneficial, and in fact, the optimal treatment may be medical therapy such as hypertension treatment to optimize afterload. However, prior studies on the medical management of HFpEF have not identified beneficial therapies in this heterogeneous population [29]. Prognostically, as we demonstrated, AFMR may be less harmful towards survival compared with MR occurring in ventricular dysfunction. Therefore, understanding the optimal treatment threshold and approach further requires mechanistic insights into the etiology of the AFMR.

Proposed treatment for AFMR is evolving with increasing mechanistic understanding of how atrial dysfunction may lead to MR [3]. A single-center observational study suggested that AFMR may be better treated with mitral annuloplasty than with replacement, owing to the isolated nature of annular dilatation in the absence of ventricular remodeling [30]. Routine catheter-based ablation or Cox-MAZE operation to treat atrial arrhythmias at the time of mitral valve operation is likely important. The role of transcatheter edge-to-edge repair for AFMR remains elusive as randomized trials on the application of MitraClip (Abbott Vascular, Menlo Park, CA) for functional MR excluded patients with AFMR [21, 22]. Identifying the optimal treatment approach for AFMR remains a topic for future investigation.

### Limitations

The small sample size of the patients with severe SMR precluded further stratification between moderate and severe MR. We demonstrated the incrementally worse survival across increasing SMR severities, and the finding likely extrapolates to the difference in moderate and severe MR with a larger sample. However, beyond prognostic significance, we could not infer differential therapeutic approach for different types of SMR. The population included those who underwent echocardiograms related to a health-system encounter and may not generalize to the general population in the community. Nevertheless, our inclusion of academic and community sites within the health-system network enabled us to capture individuals with a broad range of characteristics and likely minimized center-specific patient selection. Measurements important to further characterizing the ventricular function, such as left-ventricular end-diastolic dimension, were not consistently available and were not used in adjustment or matching in the main analysis but were descriptively reported. Inter-rater variability may exist among cardiologists who interpreted the echocardiograms. Although likely small in number, patients who relocated to outside of Connecticut and died would not have been captured by the vital statistics used in this study. We were not able to delineate the etiology of atrial dilation. This is an important limitation, as physiologic and pathologic enlargement of the left atrium may have prognostic differences [31].

## Conclusions

In this study of echocardiograms obtained across a large health system encompassing academic and community settings, patients with isolated atrial dysfunction had better survival compared with ventricular dysfunction across comparable severities of secondary mitral regurgitation. Substantially worse survival associated with mild or greater SMR in both atrial and ventricular dysfunctions warrant close monitoring at the onset of SMR irrespective of the type. Prospective evaluation of such close monitoring is needed to confirm the finding of the study.

## Supporting information

S1 Table ICD-10 code used to define comorbidities and exclusion. CABG = coronary artery bypass graft; AIDS = acquired immunodeficiency syndrome.
(DOCX)

S2 Table Estimated number of deaths with varying severities of SMR in atrial and ventricular dysfunctions. The table summarizes estimated number of deaths under constant follow-up time, had all patient had no/trace MR, mild MR, or moderate/severe MR. The magnitude of increase with increasing severities of SMR is greater in ventricular dysfunction than in atrial dysfunction.
(DOCX)

S1 Fig Propensity score density before and after matching. Density plot of the propensity score before (left) and after (right) matching. Matching was performed based on the propensity score and mitral regurgitation severity. After matching, the distribution of the propensity score became similar between atrial and ventricular dysfunction group.
(DOCX)

S2 Fig Standardized distances of patient characteristics between atrial and ventricular dysfunction before and after matching. The figure is a Love plot showing the standardized distances of each patient characteristic between atrial and ventricular dysfunction groups before and after matching. After matching, all covariates had <0.1 absolute standardized mean difference, indicating a good match. Horizontal lines were drawn every 4 variables to help visualize the corresponding marker.
(DOCX)

## References

[1] Deferm S, Bertrand PB, Verbrugge FH, et al. Atrial Functional Mitral Regurgitation: JACC Review Topic of the Week. *J Am Coll Cardiol*. 2019;73(19):2465–2476. doi: 10.1016/j.jacc.2019.02.061 31097168

[2] Delgado V, Bax JJ. Atrial Functional Mitral Regurgitation: From Mitral Annulus Dilatation to Insufficient Leaflet Remodeling. *Circ Cardiovasc Imaging*. 2017;10(3). doi: 10.1161/CIRCIMAGING.117.006239 28289022

[3] Kagiyama N, Hayashida A, Toki M, et al. Insufficient Leaflet Remodeling in Patients With Atrial Fibrillation: Association With the Severity of Mitral Regurgitation. *Circ Cardiovasc Imaging*. 2017;10(3). doi: 10.1161/CIRCIMAGING.116.005451 28289019

[4] Kagiyama N, Mondillo S, Yoshida K, Mandoli GE, Cameli M. Subtypes of Atrial Functional Mitral Regurgitation: Imaging Insights Into Their Mechanisms and Therapeutic Implications. *JACC Cardiovasc Imaging*. 2020;13(3):820–835. doi: 10.1016/j.jcmg.2019.01.040 31422123

[5] Hirji SA, Cote CL, Javadikasgari H, Malarczyk A, McGurk S, Kaneko T. Atrial functional versus ventricular functional mitral regurgitation: Prognostic implications. *J Thorac Cardiovasc Surg*. Published online December 31, 2020. doi: 10.1016/j.jtcvs.2020.12.098 33526277

[6] Okamoto C, Okada A, Nishimura K, et al. Prognostic comparison of atrial and ventricular functional mitral regurgitation. *Open Heart*. 2021;8(1). doi: 10.1136/openhrt-2021-001574 33589540 PMC7887352

[7] Baumgartner H, Falk V, Bax JJ, et al. 2017 ESC/EACTS Guidelines for the management of valvular heart disease. *Eur Heart J*. 2017;38(36):2739–2791. doi: 10.1093/eurheartj/ehx391 28886619

[8] Gertz ZM, Raina A, Saghy L, et al. Evidence of atrial functional mitral regurgitation due to atrial fibrillation: reversal with arrhythmia control. *J Am Coll Cardiol*. 2011;58(14):1474–1481. doi: 10.1016/j.jacc.2011.06.032 21939832

[9] Wu JT, Zaman JAB, Yakupoglu HY, et al. Catheter Ablation of Atrial Fibrillation in Patients With Functional Mitral Regurgitation and Left Ventricular Systolic Dysfunction. *Front Cardiovasc Med*. 2020;7:596491. doi: 10.3389/fcvm.2020.596491 33381527 PMC7767831

[10] Vohra HA, Whistance RN, Magan A, Sadeque SA, Livesey SA. Mitral valve repair for severe mitral regurgitation secondary to lone atrial fibrillation. *Eur J Cardiothorac Surg*. 2012;42(4):634–637. doi: 10.1093/ejcts/ezs029 22323495

[11] Takahashi Y, Abe Y, Sasaki Y, et al. Mitral valve repair for atrial functional mitral regurgitation in patients with chronic atrial fibrillation. *Interact Cardiovasc Thorac Surg*. 2015;21(2):163–168. doi: 10.1093/icvts/ivv119 25980774

[12] Acker MA, Parides MK, Perrault LP, et al. Mitral-Valve Repair versus Replacement for Severe Ischemic Mitral Regurgitation. *New England Journal of Medicine*. 2014;370(1):23–32. doi: 10.1056/NEJMoa1312808 24245543 PMC4128011

[13] Yale New Haven Health Systems | Locations. Accessed February 13, 2021. https://www.ynhhs.org/find-a-location/locations-and-facilities.aspx?option=type&value=Hospitals

[14] Lang RM, Badano LP, Mor-Avi V, et al. Recommendations for cardiac chamber quantification by echocardiography in adults: an update from the American Society of Echocardiography and the European Association of Cardiovascular Imaging. *J Am Soc Echocardiogr*. 2015;28(1):1–39.e14. doi: 10.1016/j.echo.2014.10.003 25559473

[15] Matsumori M, Kawashima M, Aihara T, et al. Efficacy of left atrial plication for atrial functional mitral regurgitation. *Gen Thorac Cardiovasc Surg*. 2021;69(3):458–465. doi: 10.1007/s11748-020-01483-3 32951140 PMC7900035

[16] Amabile A, Fereydooni S, Mori M, et al. Variable definitions and treatment approaches for atrial functional mitral regurgitation: A scoping review of the literature. *J Card Surg*. 2022;37(5):1182–1191. doi: 10.1111/jocs.16312 35179258

[17] Kundi H, Strom JB, Valsdottir LR, et al. Trends in Isolated Surgical Aortic Valve Replacement According to Hospital-Based Transcatheter Aortic Valve Replacement Volumes. *JACC Cardiovasc Interv*. 2018;11(21):2148–2156. doi: 10.1016/j.jcin.2018.07.002 30343022

[18] Iacus SM, King G, Porro G. Causal Inference without Balance Checking: Coarsened Exact Matching. *Political Analysis*. 2012;20(1):1–24. doi: 10.1093/pan/mpr013

[19] Zhang Z, Kim HJ, Lonjon G, Zhu Y. Balance diagnostics after propensity score matching. *Ann Transl Med*. 2019;7(1):16. doi: 10.21037/atm.2018.12.10 30788363 PMC6351359

[20] Mood C. Logistic Regression: Why We Cannot Do What We Think We Can Do, and What We Can Do About It. *European Sociological Review*. 2010;26(1):67–82. doi: 10.1093/esr/jcp006

[21] Stone GW, Lindenfeld J, Abraham WT, et al. Transcatheter Mitral-Valve Repair in Patients with Heart Failure. *N Engl J Med*. 2018;379(24):2307–2318. doi: 10.1056/NEJMoa1806640 30280640

[22] Obadia JF, Messika-Zeitoun D, Leurent G, et al. Percutaneous Repair or Medical Treatment for Secondary Mitral Regurgitation. *N Engl J Med*. 2018;379(24):2297–2306. doi: 10.1056/NEJMoa1805374 30145927

[23] Abe Y, Akamatsu K, Ito K, et al. Prevalence and Prognostic Significance of Functional Mitral and Tricuspid Regurgitation Despite Preserved Left Ventricular Ejection Fraction in Atrial Fibrillation Patients. *Circ J*. 2018;82(5):1451–1458. doi: 10.1253/circj.CJ-17-1334 29553091

[24] Saito C, Minami Y, Arai K, et al. Prevalence, clinical characteristics, and outcome of atrial functional mitral regurgitation in hospitalized heart failure patients with atrial fibrillation. *J Cardiol*. 2018;72(4):292–299. doi: 10.1016/j.jjcc.2018.04.002 29752195

[25] Grigioni F, Detaint D, Avierinos JF, Scott C, Tajik J, Enriquez-Sarano M. Contribution of ischemic mitral regurgitation to congestive heart failure after myocardial infarction. *J Am Coll Cardiol*. 2005;45(2):260–267. doi: 10.1016/j.jacc.2004.10.030 15653025

[26] Writing Committee Members Otto CM, Nishimura RA, et al. 2020 ACC/AHA Guideline for the Management of Patients With Valvular Heart Disease: Executive Summary: A Report of the American College of Cardiology/American Heart Association Joint Committee on Clinical Practice Guidelines. *J Am Coll Cardiol*. 2021;77(4):450–500. doi: 10.1016/j.jacc.2020.11.035 33342587

[27] Mori M, Weininger G, Agarwal R, et al. Survival of patients with mild secondary mitral regurgitation with and without mild tricuspid regurgitation. *Can J Cardiol*. Published online June 10, 2021. doi: 10.1016/j.cjca.2021.06.005 34119634

[28] Sannino A, Smith RL, Schiattarella GG, Trimarco B, Esposito G, Grayburn PA. Survival and Cardiovascular Outcomes of Patients With Secondary Mitral Regurgitation: A Systematic Review and Meta-analysis. *JAMA Cardiol*. 2017;2(10):1130–1139. doi: 10.1001/jamacardio.2017.2976 28877291 PMC5710448

[29] Bartko PE, Hülsmann M, Hung J, et al. Secondary valve regurgitation in patients with heart failure with preserved ejection fraction, heart failure with mid-range ejection fraction, and heart failure with reduced ejection fraction. *Eur Heart J*. 2020;41(29):2799–2810. doi: 10.1093/eurheartj/ehaa129 32350503 PMC8453270

[30] Carino D, Lapenna E, Ascione G, et al. Is mitral annuloplasty an effective treatment for severe atrial functional mitral regurgitation? *J Card Surg*. 2021;36(2):596–602. doi: 10.1111/jocs.15273 33386760

[31] Chen YC, Voskoboinik A, Gerche AL, Marwick TH, McMullen JR. Prevention of Pathological Atrial Remodeling and Atrial Fibrillation: JACC State-of-the-Art Review. *J Am Coll Cardiol*. 2021;77(22):2846–2864. doi: 10.1016/j.jacc.2021.04.012 34082914

