# Supplementary material for: Impact of secondary mitral regurgitation on survival in atrial and ventricular dysfunction
Source: PLoS One. 2022 Dec 22;17(12):e0277385. doi: 10.1371/journal.pone.0277385 (PMC9778994; doi:10.1371/journal.pone.0277385)
Supplement: S1 Table — CABG = coronary artery bypass graft; AIDS = acquired immunodeficiency syndrome. (DOCX) [file pone.0277385.s001.docx]

Supplementary Table 1: ICD-10 code used to define comorbidities and exclusion

| **Variables** | **ICD-10 code** |
| --- | --- |
| Diabetes | E10.0-E10.9, E11.0-E11.9, E12.0-E12.9, E13.0-E13.9, E14.0-14.9 |
| Myocardial Infarction | I21.x-I23.x |
| Heart failure | I50.x |
| Prior CABG | Z95.1 |
| Valve surgery | Z95.2-Z95.4 |
| Atrial fibrillation/flutter | I48.x |
| Hypertension | I10.x-I13.x, I15.x, I16.x |
| Peripheral vascular disorders | I70.x, I71.x, I73.1, I73.8, I73.9, I77.1, I79.0, I79.2, K55.1, K55.8, K55.9, Z95.8, Z95.9 |
| Cerebrovascular disorders | G45.x, G46.x, H34.0, I60.x-I69.x |
| Chronic obstructive pulmonary disease | I27.8, 127.9, J40.x-J47.x, J60.x-J67.x, J68.4, J70.1, J70.3 |
| Liver disease | B18.x, I85.0, I85.9, I86.4, I98.2, K70.0-K70.4, K70.9, K71.1, K71.3-K71.5, K71.7, K72.1, K72.9, K73.x, K74.x, K76.0, K76.2-K76.9. Z94.4 |
| Renal failure | I12.0, I13.1, N03.2-N03.7, N05.2-N05.7, N18.x, NI9.x, N25.0, Z49.0-Z49.2, Z94.0, Z99.2 |
| Anemia | D50.x |
| Rheumatoid disorders | M05.x, M06.x, M31.5, M32.x-M34.x, M35.1, M35.3, M36.0 |
| Peptic ulcer disease | K25.x-K28.x |
| Dementia | F00.x-F03.x, F05.1, G30.x, G31.1 |
| Depression | F20.4, F31.3-F31.5, F32.x, F33.x, F34.1, F41.2, F43.2 |
| Cancer | C00.x-C26.x, C30.x-C34.x, C37.x-C41.x, C43.x, C45.x-C58.x, C60.x-C85.x, C88.x, C90.x-C97.x |
| Substances use | E52.x, F10.x-F16.x, F18.x, F19.x, G62.1, I42.6, K29.2, K70.0, K70.3, K70.9, T51.x, Z50.2, Z71.4, Z71.5, Z72.1, Z72.2 |
| AIDS | B20.x-B22.x, B24.x |

CABG = coronary artery bypass graft; AIDS = acquired immunodeficiency syndrome
