# Supplementary material for: Impact of secondary mitral regurgitation on survival in atrial and ventricular dysfunction
Source: PLoS One. 2022 Dec 22;17(12):e0277385. doi: 10.1371/journal.pone.0277385 (PMC9778994; doi:10.1371/journal.pone.0277385)
Supplement: S2 Table — The table summarizes estimated number of deaths under constant follow-up time, had all patient had no/trace MR, mild MR, or moderate/severe MR. The magnitude of increase with increasing severities of SMR is greater in ventricular dysfunction than in atrial dysfunction. (DOCX) [file pone.0277385.s002.docx]

Supplemental Table 2: Estimated number of deaths with varying severities of SMR in atrial and ventricular dysfunctions

|  | Atrial dysfunction | |  | Ventricular dysfunction | |
| --- | --- | --- | --- | --- | --- |
| MR severity | Expected death (n) | % change from No/trace MR |  | Expected death (n) | % change from No/trace MR |
| No/trace | 906 | - |  | 680 | - |
| Mild | 962 | 6% |  | 1010 | 49% |
| Moderate/severe | 1179 | 30% |  | 1345 | 98% |

The table summarizes estimated number of deaths under constant follow-up time, had all patient had no/trace MR, mild MR, or moderate/severe MR. The magnitude of increase with increasing severities of SMR is greater in ventricular dysfunction than in atrial dysfunction.
