# Supplementary material for: Impact of secondary mitral regurgitation on survival in atrial and ventricular dysfunction
Source: PLoS One. 2022 Dec 22;17(12):e0277385. doi: 10.1371/journal.pone.0277385 (PMC9778994; doi:10.1371/journal.pone.0277385)
Supplement: S1 Fig — Density plot of the propensity score before (left) and after (right) matching. Matching was performed based on the propensity score and mitral regurgitation severity. After matching, the distribution of the propensity score became similar between atrial and ventricular dysfunction group. (DOCX) [file pone.0277385.s003.docx]

Supplemental Figure 1: Propensity score density before and after matching


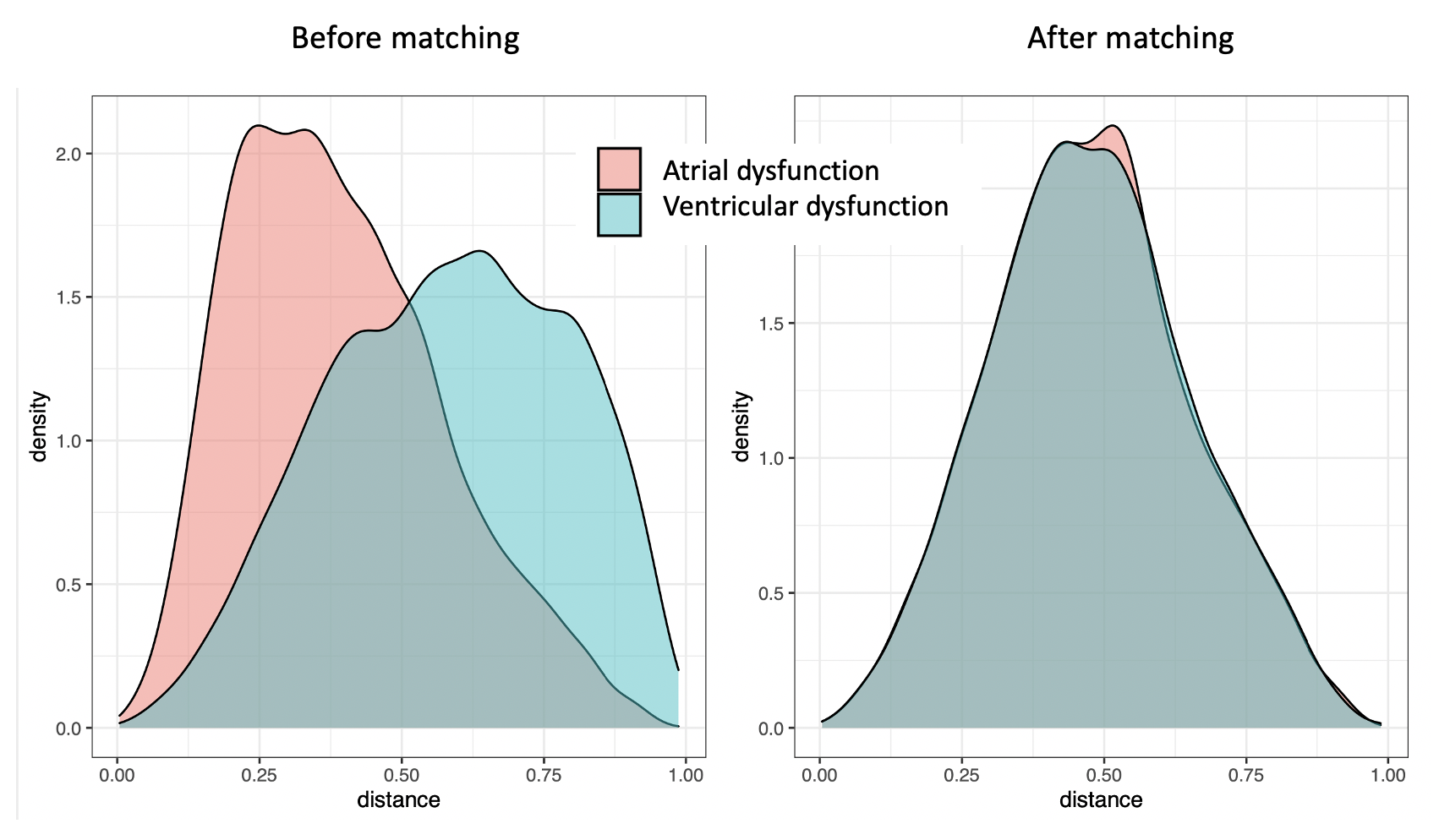


Density plot of the propensity score before (left) and after (right) matching. Matching was performed based on the propensity score and mitral regurgitation severity. After matching, the distribution of the propensity score became similar between atrial and ventricular dysfunction group.
