# Supplementary material for: Impact of secondary mitral regurgitation on survival in atrial and ventricular dysfunction
Source: PLoS One. 2022 Dec 22;17(12):e0277385. doi: 10.1371/journal.pone.0277385 (PMC9778994; doi:10.1371/journal.pone.0277385)
Supplement: S2 Fig — The figure is a Love plot showing the standardized distances of each patient characteristic between atrial and ventricular dysfunction groups before and after matching. After matching, all covariates had <0.1 absolute standardized mean difference, indicating a good match. Horizontal lines were drawn every 4 variables to help visualize the corresponding marker. (DOCX) [file pone.0277385.s004.docx]

Supplemental Figure 2: Standardized distances of patient characteristics between atrial and ventricular dysfunction before and after matching


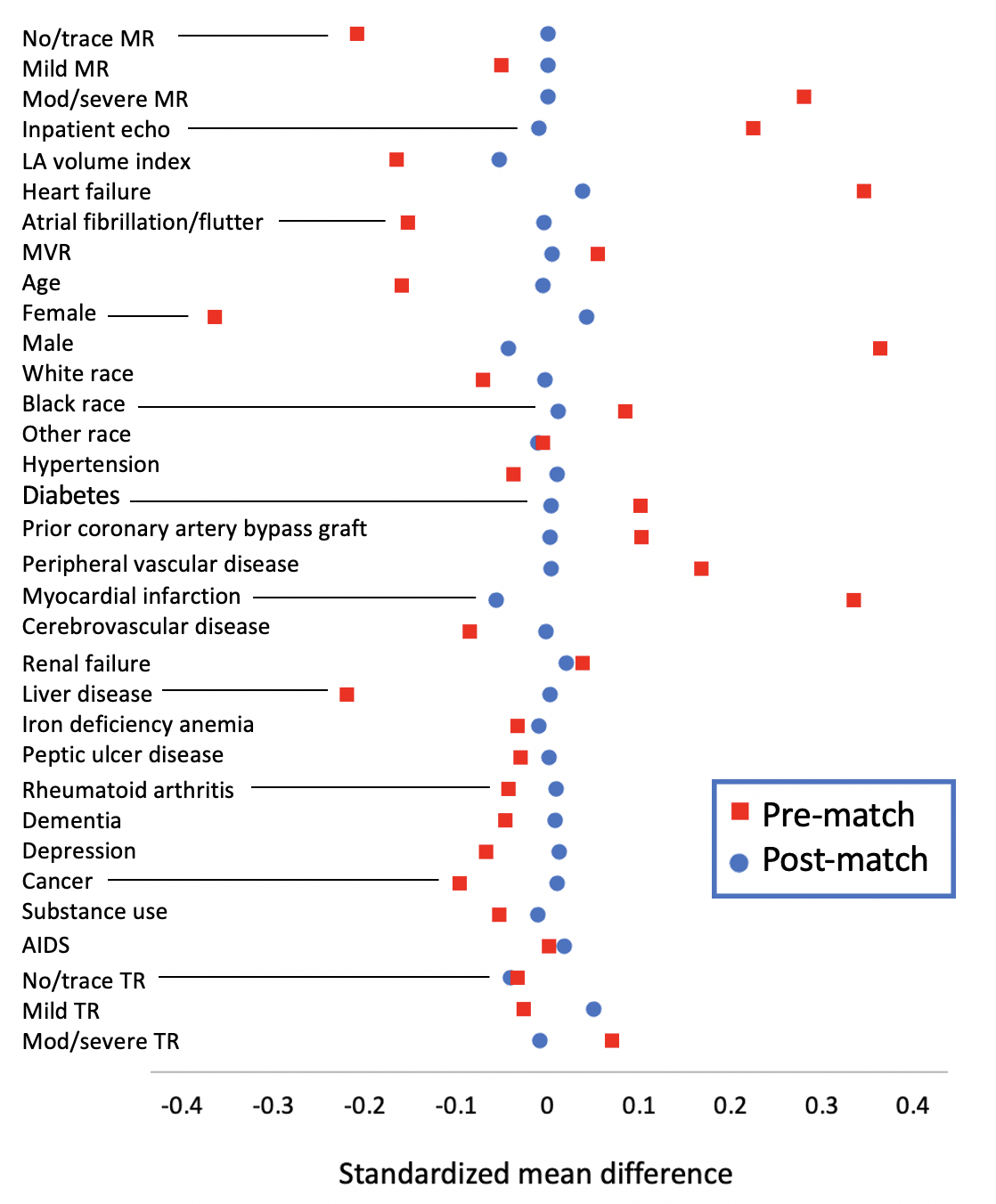


The figure is a Love plot showing the standardized distances of each patient characteristic between atrial and ventricular dysfunction groups before and after matching. After matching, all covariates had <0.1 absolute standardized mean difference, indicating a good match. Horizontal lines were drawn every 4 variables to help visualize the corresponding marker.
